# Comprehensive analysis of SYTL4 differential expression and clinical implications in DLBCL

**DOI:** 10.3389/fgene.2026.1908683

**Published:** 2026-08-18

**Authors:** Yue Li, Qiqi Zhou

**Affiliations:** Department of Cardio-Pulmonary Function, Tianjin Medical University Cancer Institute and Hospital, National Clinical Research Center for Cancer, Tianjin’s Clinical Research Center for Cancer, Key Laboratory of Cancer Prevention and Therapy, Tianjin, China

**Keywords:** DLBCL, prognostic biomarker, RNA-seq, SYTL4, TMA

## Abstract

**Background:**

Diffuse large B-cell lymphoma (DLBCL), a common type of non-Hodgkin lymphoma (NHL), is associated with heterogeneous clinical outcomes, with a subset of patients experiencing refractory disease or relapse, underscoring the need for novel biomarkers to refine risk stratification. This study aimed to investigate the biological role and clinical significance of synaptotagmin-like 4 (SYTL4) in DLBCL.

**Methods:**

RNA-seq data from TCGA and GTEx were normalized (TPM) and log2-transformed. Survival analysis stratified patients based on the median SYTL4 expression level (cutoff = 0.304), with Kaplan-Meier curves and Cox regression analysis performed. Differentially expressed genes (|log2FC|>1, FDR<0.05) underwent functional annotation (GOKEGG, GSEA) and PPI network constructed. Immune infiltration was assessed via ssGSEA. Formalin-fixed paraffin-embedded (FFPE) tissue blocks from testicular DLBCL patients were obtained. A tissue microarray was constructed. Immunohistochemistry with anti-SYTL4 antibody was performed, and digital images were scored semi-quantitatively (0–3+) by two pathologists.

**Results:**

RNA-seq analysis revealed a significant upregulation of SYTL4 expression in DLBCL patients compared to healthy donors, observed in both tissue and peripheral blood samples, a finding further validated by immunohistochemical staining. Survival analysis revealed that elevated SYTL4 expression was correlated with an unfavorable prognosis (P = 0.037), and multivariate Cox regression confirmed that high SYTL4 expression was an independent prognostic factor (HR = 25.541, P = 0.017). Differential gene screening identified 1,074 differentially expressed genes, including 628 upregulated genes and 446 downregulated genes. Enrichment analysis revealed a significant association of SYTL4-related genes with pathways involved in cell–cell adhesion and the immune response. Protein‒protein interaction (PPI) network analysis revealed interactions between SYTL4 and key regulators such as phosphatase and actin regulator 2 (PHACTR2), suggesting its potential role in DLBCL progression.

**Conclusion:**

These findings highlight the potential of SYTL4 as a promising diagnostic marker, prognostic biomarker, and therapeutic target. Further exploration of its applicability in immunotherapy may lead to the development of personalized treatment strategies for DLBCL patients.

## Introduction

Diffuse large B-cell lymphoma (DLBCL), a predominant type of non-Hodgkin lymphoma (NHL), continues to demonstrate increasing global incidence rates accompanied by substantial mortality burdens, imposing significant clinical and socioeconomic challenges (Friedberg and Fisher, 2008). While current therapeutic strategies encompass multimodal approaches, including chemotherapy, radiotherapy, targeted therapies, and immunotherapeutic interventions, persistent limitations in treatment efficacy, acquired drug resistance mechanisms, and suboptimal clinical outcomes in refractory cases remain critical concerns. These unresolved challenges underscore the imperative for advancing early diagnostic modalities and developing personalized therapeutic paradigms (Ni et al., 2016). The molecular pathogenesis of DLBCL has garnered increasing research attention, with comprehensive gene expression profiling emerging as a pivotal tool for elucidating tumor heterogeneity and identifying prognostic biomarkers. This genomic characterization enables refined molecular stratification, potentially informing precision medicine approaches to optimize therapeutic decision-making (Li et al., 2018).

Synaptotagmin-like 4 (SYTL4), a regulator of dense-core granule exocytosis and secretion of hormones in the pancreas and pituitary, has recently emerged as a cancer-associated protein with dysregulated expression patterns across diverse malignancies (Miller and Grunewald, 2015). SYTL4 has been implicated in regulating exosome secretion, potentially influencing tumor microenvironment interactions (Liao et al., 2020). In triple-negative breast cancer (TNBC), SYTL4 overexpression correlates with chemoresistance and poor prognosis, and mechanistically regulates microtubule stability and exosome secretion (Liao et al., 2020; Liu et al., 2020). In acute myeloid leukemia (AML), elevated SYTL4 expression is significantly associated with adverse clinical outcomes, supporting its potential as a prognostic biomarker (Xie and Wei, 2024). In colonic adenocarcinoma, SYTL4 expression signatures have been incorporated into prognostic models, with preliminary evidence indicating its involvement in immune microenvironment modulation (Xu et al., 2024). However, the molecular role and therapeutic implications of SYTL4 in DLBCL remain unexplored, highlighting the novelty and rationale of this study.

This study integrates RNA-sequencing data from The Cancer Genome Atlas (TCGA) cohort (n = 10,534) and normal tissue data from the Genotype-Tissue Expression (GTEx) project (n = 7,568), along with the corresponding clinical patient data. Through systematic bioinformatics analysis, we investigated the expression profile and biological implications of SYTL4 and its cofunction genes in DLBCL. We aimed to elucidate the interrelationships between SYTL4 and other critical genes, along with their potential mechanistic roles in DLBCL progression, thereby establishing a foundation for subsequent experimental validation and clinical applications. Through this exploration, we anticipate providing new perspectives for DLBCL treatment strategies to promote the development of personalized medicine.

## Materials and methods

### Data acquisition and preprocessing

RNA-seq data for 33 TCGA cancer types and matched GTEx normal tissues were obtained from University of California Santa Cruz XENA (UCSC XENA, https://xenabrowser.net), with raw counts converted to transcripts per million (TPM) values via Toil pipelines (Vivian et al., 2017). The DLBCL-specific RNA-seq data (STAR-generated counts) and clinicopathological records of 48 patients were downloaded from the TCGA portal (Liu et al., 2018). In addition, we incorporated the GSE83632 (Azzaoui et al., 2016) dataset from Gene Expression Omnibus (GEO, https://www.ncbi.nlm.nih.gov/geo/) for analysis. This dataset contains whole-transcriptome expression data derived from peripheral blood samples of 76 DLBCL patients and 87 healthy donors. All expression values were log2 (value + 1) transformed.

### Survival analysis

Patients were stratified into SYTL4 high- and SYTL4 low-expression groups on the basis of the median SYTL4 mRNA expression level (cutoff = 0.304, 24 vs. 24). Survival curves were generated via the survival package (v3.3.1), with the log-rank test used to assess intergroup differences at a significance threshold of *P* < 0.05.

Univariate Cox analysis was used to screen variables with P < 0.10 (Jiang et al., 2022) for multivariate modeling via stepwise regression.

The nomogram for predicting 1-year, 3-year, and 5-year survival rates was developed with the rms package (v6.3-0) and was calibrated with 1,000 bootstrap resamples.

The predictive accuracy of SYTL4 expression and the nomogram model for 1-, 3-, and 5-year survival rates was assessed via the time-dependent receiver operating characteristic (timeROC) package (v0.4). Time-dependent area under the curve (AUC) values were calculated via the Kaplan‒Meier weighting method, with confidence intervals estimated by 200 bootstrap resamples (Heagerty et al., 2000).

### Differentially expressed genes (DEGs) analysis

DLBCL patients were stratified into SYTL4 high-expression and SYTL4 low-expression groups on the basis of the median SYTL4 mRNA expression level (cutoff = 0.304). Differential analysis was performed via DESeq2 (v1.36.0) (Love et al., 2014) and edgeR (v3.38.2) (Robinson et al., 2010) with thresholds of |log2FC| > 1 and a false discovery rate (FDR) < 0.05.

### Gene enrichment analysis

Gene Ontology (GO) and Kyoto Encyclopedia of Genes and Genomes (KEGG) pathway (Kanehisa and Goto, 2000; Kanehisa, 2019; Kanehisa et al., 2025) analyses were conducted via clusterProfiler (v4.4.4) (Yu et al., 2012). Significantly enriched terms were defined as those with an FDR<0.05, with z scores calculated on the basis of up/downregulation trends of differentially expressed genes.

Using the c2.cp.all.v2022.1.Hs.symbols.gmt gene set from the Molecular Signatures Database (MSigDB) (https://www.gsea-msigdb.org), gene set enrichment analysis (GSEA) was performed with a weighted scoring algorithm. The gene set size ranged from 15 to 500; the significance threshold was an FDR<0.25 (Broad Institute standards) (Subramanian et al., 2005).

### Immune infiltration analysis

Single-sample GSEA (ssGSEA) was used to quantify immune cell infiltration levels via 24 immune cell markers (Bindea et al., 2013). Pearson correlation was used to assess SYTL4-immune cell relationships, and the Wilcoxon rank-sum test was used to compare intergroup differences.

### Protein‒protein interactions (PPIs)

The PPI network centered on SYTL4 was constructed via the STRING database (https://string-db.org). DEGs were filtered with thresholds of |log2FC| > 4 and adjusted p value (p.adj) < 0.05. The network parameters were configured with a minimum required interaction score of 0.40 and a maximum number of interactors of no more than 5.

### Tissue samples

Formalin-fixed paraffin-embedded (FFPE) tissue blocks were obtained from the Biobank of Tianjin Medical University Cancer Institute and Hospital. The use of all human tissues was approved by the Ethics Committee of Tianjin Medical University Cancer Institute and Hospital (Approval No.: E20250747) and complied with the Declaration of Helsinki.

### Tissue microarray (TMA) construction

We collected tissue blocks from patients with testicular DLBCL who were treated at Tianjin Medical University Cancer Institute and Hospital between March 2014 and December 2020. Using a fully automated tissue microarrayer (TMAGM: TMA Grand Master, Epredia, US), tissue cores with a diameter of 2.0 mm were extracted from donor paraffin blocks and transferred into a recipient block to construct a TMA block for immunochemical analysis. The final TMA block contained 16 tissue cores, including 14 tumor tissue samples and 2 adjacent normal tissue samples serving as controls.

### Immunohistochemistry (IHC)

Sections of 4 μm thickness were sectioned from the TMA block and mounted on charged glass slides. Immunohistochemical staining was performed using a standard protocol. Briefly, the slides were deparaffinized in xylene, rehydrated through a graded ethanol series, and antigen retrieval was performed by microwave heating in 10 mM citrate buffer (pH 6.0) for 10 min. Endogenous peroxidase activity was blocked with 3% hydrogen peroxide, and nonspecific binding were blocked with 5% bovine serum albumin (BSA) at room temperature for 30 min. The slides were incubated overnight at 4 °C with the primary antibody, SYTL4 Polyclonal Antibody (PA5-35992, 1:1,000, Invitrogen, US). After washing with phosphate buffer saline (PBS), the slides were incubated with an HRP-labeled secondary antibody at room temperature for 30 min. And diaminobenzidine (DAB) was the chromogen. Finally, slides were counterstained with hematoxylin, dehydrated, cleared, and mounted with a synthetic resin.

### Digital imaging and evaluation

Stained slides were scanned using a digital slide scanner (Aperio AT2, Leica, Germany) to obtain high-resolution digital images. Two pathologists independently assessed the staining results based on a semi-quantitative score (0–3+) combining staining intensity and percentage of positive cells. Discrepant evaluations were resolved by consensus.

### Statistical analysis

The normality of continuous variables was tested via the Shapiro‒Wilk test; group comparisons were performed via t tests or Wilcoxon rank‒sum tests. Categorical variables were analyzed by the chi-square test or Fisher’s exact test. Multiple testing correction was applied via the Benjamini‒Hochberg method.

## Results

### Specific upregulation of SYTL4 in DLBCL and its diagnostic value

**FIGURE 1:**
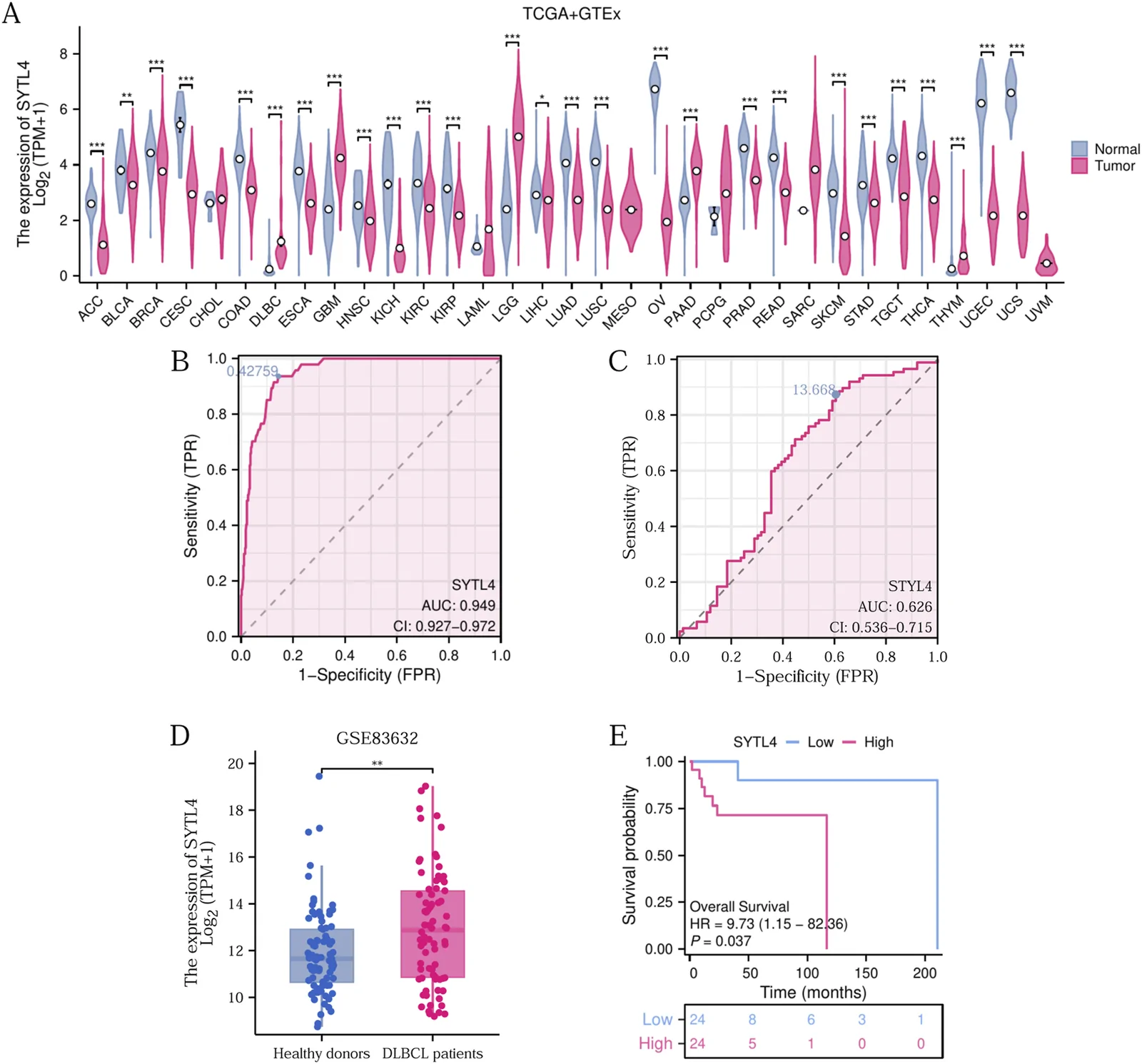
Expression level of SYTL4 exhibits good diagnostic and prognostic value for patients with DLBCL. **(A)** Comparative analysis of SYTL4 mRNA expression levels in tumor and adjacent normal tissues across various malignancies. **(B)** The diagnostic predictive value of SYTL4 expression level was assessed using ROC curve analysis in TCGA/GTEx tissue cohort. **(C)** The diagnostic predictive value of SYTL4 expression level was assessed using ROC curve analysis in the GSE83632 peripheral blood cohort. **(D)** Comparative analysis of SYTL4 mRNA expression levels in peripheral blood of DLBCL patients and healthy donors (GSE83632). **(E)** Kaplan-Meier curve illustrating the comparison of overall survival between subgroups of patients with DLBCL with a high/low SYTL4 mRNA expression. *P < 0.05; **P < 0.01; ***P < 0.001. SYTL4, synaptotagmin-like 4; DLBCL, diffuse large B-cell lymphoma; GBM, glioblastoma multiforme; LGG, brain lower grade glioma; PAAD, pancreatic adenocarcinoma; THYM, thymoma; ACC, adrenocortical carcinoma; BLCA, bladder urothelial carcinoma; BRCA, breast invasive carcinoma; CESC, cervical squamous cell carcinoma; COAD, colon adenocarcinoma; ESCA, esophageal carcinoma; HNSC, head and neck squamous cell carcinoma; KICH, kidney chromophobe; KIRC, kidney renal clear cell carcinoma; KIRP, kidney renal papillary cell carcinoma; LIHC, liver hepatocellular carcinoma; LUAD, lung adenocarcinoma; LUSC, lung squamous cell carcinoma; OV, ovarian serous cystadenocarcinoma; PRAD, prostate adenocarcinoma; READ, rectum adenocarcinoma; SKCM, skin cutaneous melanoma; STAD, stomach adenocarcinoma; TGCT, testicular germ cell tumors; THCA, thyroid carcinoma; UCEC, uterine corpus endometrial carcinoma; UCS, uterine carcinosarcoma; AUC, area under the curve; CI, confidence intervals; HR, hazard ratio; FPR, false positive rate; TPR, true positive rate.

Pan-cancer analysis revealed tissue-specific dysregulation of SYTL4 across tumor types (Figure 1A). SYTL4 was significantly upregulated in DLBCL and four other cancers, glioblastoma multiforme (GBM), brain lower grade glioma (LGG), pancreatic adenocarcinoma (PAAD), and thymoma (THYM), while downregulated in multiple other malignancies (full expression landscape is shown in Figure 1A). Consistent with this, SYTL4 upregulation was also observed in DLBCL patients from the independent GSE83632 cohort, based on peripheral blood transcriptome data (Figure 1D).

### Diagnostic value of SYTL4 in DLBCL across tissue and peripheral blood cohorts

In TCGA/GTEx tissue cohort, receiver operating characteristic (ROC) curve analysis (Figure 1B) showed an AUC of 0.949 (95% confidence interval [CI]: 0.927–0.972), with an optimal cutoff value of 0.428 for distinguishing DLBCL tumors from normal tissues. These results suggest the potential of SYTL4 as a tissue-based diagnostic marker for DLBCL. However, when evaluated in the GSE83632 peripheral blood cohort, the discriminatory ability was substantially lower (AUC = 0.626; 95% CI: 0.536–0.715, Figure 1C), indicating limited diagnostic value in blood samples.

### Associations between SYTL4 expression and the clinicopathological features of patients with DLBCL

The baseline demographic and clinical characteristics of DLBCL patients stratified by the median SYTL4 mRNA level (cutoff = 0.304) are summarized in Table 1. Additionally, the SYTL4 high-expression group presented a greater mortality rate (29.2% vs. 8.3%) than did the SYTL4 low-expression group. While no statistically significant differences were observed in conventional parameters such as clinical stage, extranodal involvement, or primary therapy outcome, a trend toward female predominance was identified in the SYTL4 high-expression group (F vs. M: 62.5% vs. 45.8%), although statistical significance was not reached.

**TABLE 1:** Association between SYTL4 and clinicopathological features of patients with DLBCL.

Characteristics	Low expression of SYTL4	High expression of SYTL4	P value
n	24	24	​
Clinical stage, n (%)	​	​	0.952
Stage I&Stage II	12 (60%)	13 (59.1%)	​
Stage III&Stage IV	8 (40%)	9 (40.9%)	​
Gender, n (%)	​	​	0.247
Female	11 (45.8%)	15 (62.5%)	​
Male	13 (54.2%)	9 (37.5%)	​
Race, n (%)	​	​	0.768
White	14 (58.3%)	15 (62.5%)	​
Asian and Black or African American	10 (41.7%)	9 (37.5%)	​
Age, n (%)	​	​	0.771
<= 60	13 (54.2%)	14 (58.3%)	​
>60	11 (45.8%)	10 (41.7%)	​
Weight, n (%)	​	​	0.771
<= 70	13 (54.2%)	14 (58.3%)	​
>70	11 (45.8%)	10 (41.7%)	​
Height, n (%)	​	​	0.562
<= 165	12 (50%)	14 (58.3%)	​
>165	12 (50%)	10 (41.7%)	​
BMI, n (%)	​	​	0.773
<= 25	12 (50%)	13 (54.2%)	​
>25	12 (50%)	11 (45.8%)	​
Extranodal involvement, n (%)	​	​	0.979
No	12 (54.5%)	13 (54.2%)	​
Yes	10 (45.5%)	11 (45.8%)	​
Primary therapy outcome, n (%)	​	​	1.000
CR&PR	20 (83.3%)	18 (81.8%)	​
PD&SD	4 (16.7%)	4 (18.2%)	​
OS event, n (%)	​	​	0.139
Alive	22 (91.7%)	17 (70.8%)	​
Dead	2 (8.3%)	7 (29.2%)	​

SYTL4, synaptotagmin-like 4; DLBCL, diffuse large B-cell lymphoma; BMI, body mass index; CR, complete response; PR, partial response; PD, progressive disease; SD, stable disease; OS, overall survival.

### Univariate and multivariate survival analysis of SYTL4 in DLBCL

K‒M analysis showed poorer overall survival (OS) in DLBCL patients with high SYTL4 expression (*P* = 0.037, Figure 1E), with a HR of 9.73 (95% CI: 1.15–82.36). Univariate Cox regression revealed three risk factors (*P* < 0.10, Table 2): primary therapy outcome progressive disease (PD): HR = 14.461, 95% CI: 2.803–74.603; *P* = 0.001); race (nonwhites (Asian and Black or African American): HR = 5.354, 95% CI: 0.974–29.450; *P* = 0.054); and SYTL4 expression (high expression: HR = 9.734, 95% CI: 1.150–82.360; *P* = 0.037). In multivariate analysis, high SYTL4 expression was independently associated with worse OS (HR = 25.541, 95% CI: 1.776–367.264; P = 0.017), along with stable disease (SD) (HR = 85.826, 95% CI: 2.792–2638.203; P = 0.011), PD (HR = 65.115, 95% CI: 3.835–1105.630; P = 0.004), and nonwhites (HR = 33.649, 95% CI: 1.800–629.132; P = 0.019).

**TABLE 2:** SYTL4 prognostic value in DLBCL assessed via univariate/multivariate Cox analyses.

Characteristics	Total (N)	Univariate analysis		Multivariate analysis	
		HR (95% CI)	P value	HR (95% CI)	P value
Primary therapy outcome	46	​	​	​	​
CR	35	Reference	​	Reference	​
PR	3	0.000 (0.000–Inf)	0.999	0.000 (0.000–Inf)	0.999
SD	3	5.055 (0.521–49.090)	0.162	85.826 (2.792–2638.203)	0.011
PD	5	14.461 (2.803–74.603)	0.001	65.115 (3.835–1105.630)	0.004
Race	48	​	​	​	​
White	29	Reference	​	Reference	​
Asian and Black or African American	19	5.354 (0.974–29.450)	0.054	33.649 (1.800–629.132)	0.019
SYTL4	48	​	​	​	​
Low	24	Reference	​	Reference	​
High	24	9.734 (1.150–82.360)	0.037	25.541 (1.776–367.264)	0.017

SYTL4, synaptotagmin-like 4; DLBCL, diffuse large B-cell lymphoma; CR, complete response; PR, partial response; PD, progressive disease; SD, stable disease; HR, hazard ratio; CI, confidence intervals.

**FIGURE 2:**
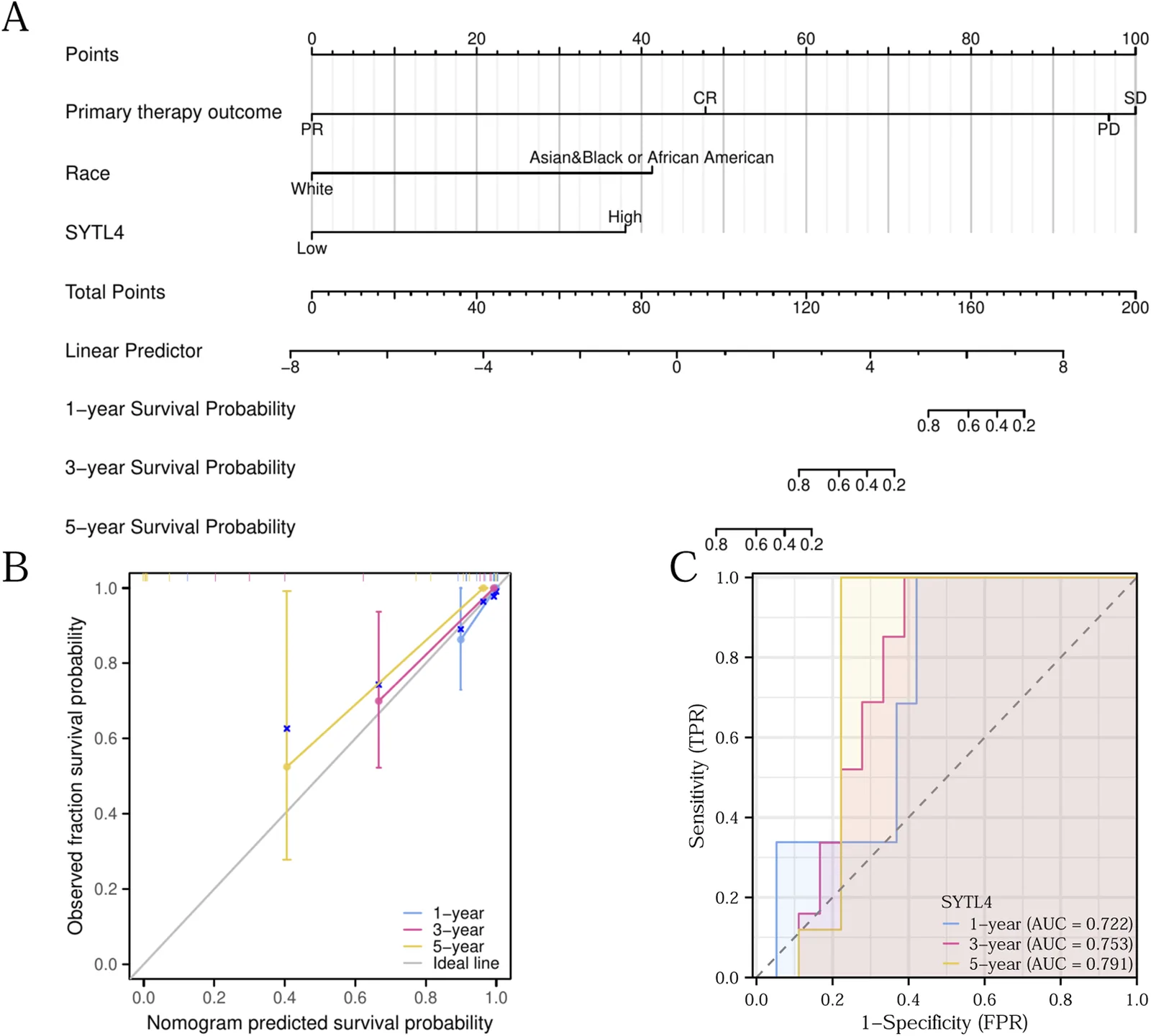
Overall survival. The construction of a nomogram curve was performed to predict the probability of OS rate of in patients at 1-, 3-, and 5-year. **(A)** The predictive factors encompassed primary therapy outcome, race and SYTL4 expression. **(B)** The nomogram enabled accurate prediction of 1-, 3-, and 5-year overall survival (OS) rates in DLBCL patients. **(C)** 1-, 3-, and 5-year timeROC curve. SYTL4, synaptotagmin-like 4; CR, complete response; PR, partial response; PD, progressive disease; SD, stable disease; AUC, area under the curve; FPR, false positive rate; TPR, true positive rate.

A nomogram was developed to predict OS at 1, 3, and 5 years based on SYTL4 expression, race, and primary therapy outcome (Figure 2A). Bias-adjusted calibration plots showed that the predicted survival probabilities were generally consistent with the observed outcomes across the follow-up period (Figure 2B). Time-dependent ROC curve analysis showed AUC values of 0.722, 0.753, and 0.791 for 1-, 3-, and 5-year OS predictions, respectively (Figure 2C), indicating modest improvement in discriminative ability over longer follow-up.

### DEGs between the SYTL4 high- and low-expression groups

**FIGURE 3:**
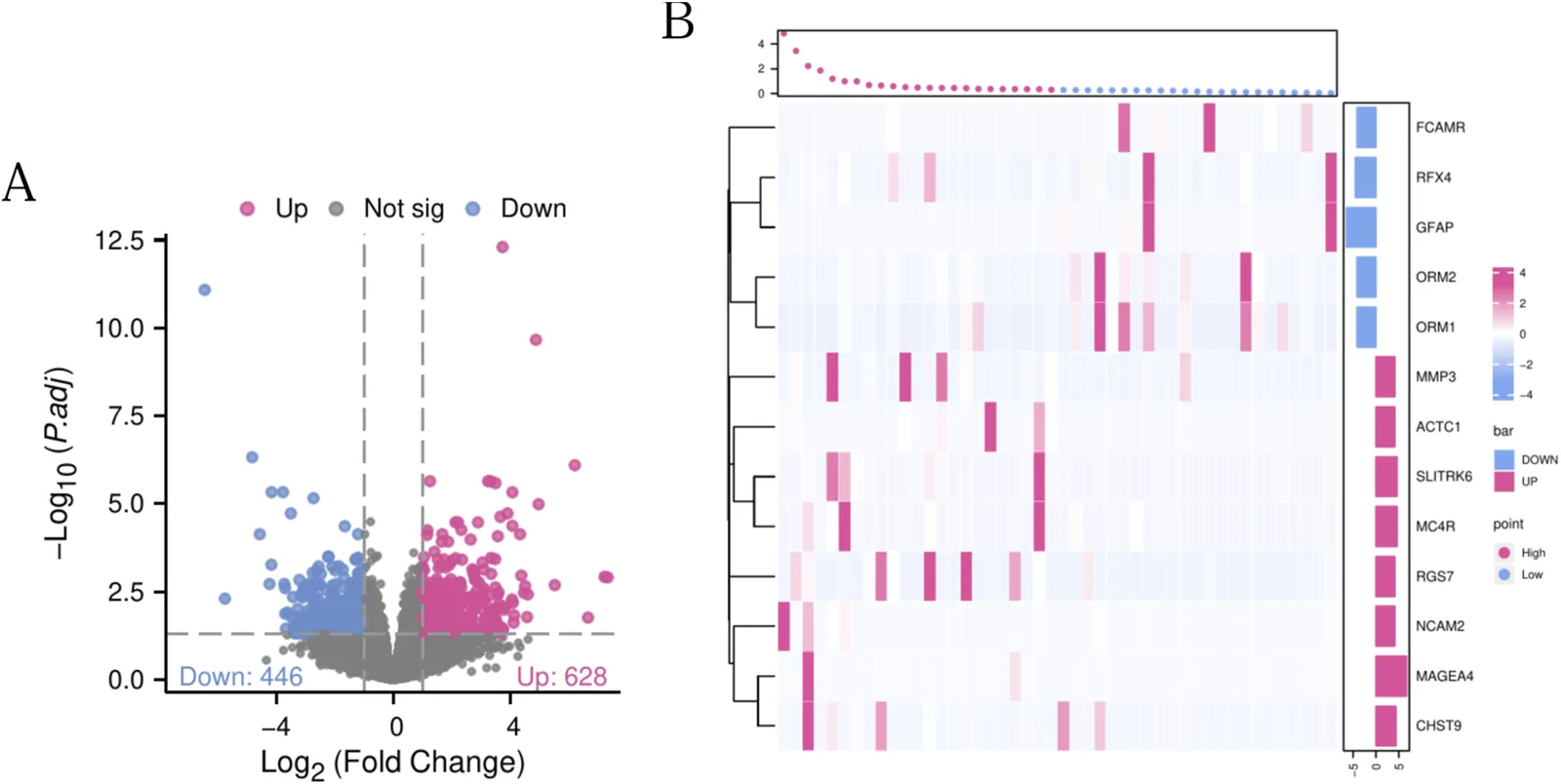
Identification of differentially expressed genes (DEGs) between the SYTL4 high- and low-expression groups. **(A)** A volcanic map depicting the DEGs. **(B)** Heatmap showing SYTL4 and its co-expressed mRNA. SYTL4, synaptotagmin-like 4; FCAMR, Fc alpha and mu receptor; RFX4, regulatory factor X4; GFAP, glial fibrillary acidic protein; ORM2, orosomucoid 2; ORM1, orosomucoid 1; MMP3, matrix metallopeptidase 3; ACTC1, actin alpha cardiac muscle 1; SLITRK6, SLIT and NTRK like family member 6; MC4R, melanocortin 4 receptor; RGS7, regulator of G protein signaling 7; NCAM2, neuralcelladhesionmolecule2; MAGEA4, melanoma-associated antigen 4; CHST9, carbohydrate sulfotransferase 9.

RNA-seq analysis of 48 DLBCL samples stratified by SYTL4 expression levels identified 1,074 DEGs meeting the threshold criteria (|log2FC| > 1, p.adj < 0.05), with 628 genes upregulated and 446 downregulated in SYTL4 high-expression tumors (Figure 3A). A stringent secondary filter (|log2FC| > 4, p.adj < 0.05) revealed that 13 genes exhibited extreme expression divergence, including eight genes (MMP3, ACTC1, SLITRK6, MC4R, RGS7, NCAM2, MAGEA4, and CHST9) that strongly positively correlated with SYTL4 expression and five genes (FCAMR, RFX4, GFAP, ORM2, and ORM1) that presented inverse correlation patterns (Figure 3B).

### The role of SYTL4 in DLBCL was investigated through gene enrichment analysis

**FIGURE 4:**
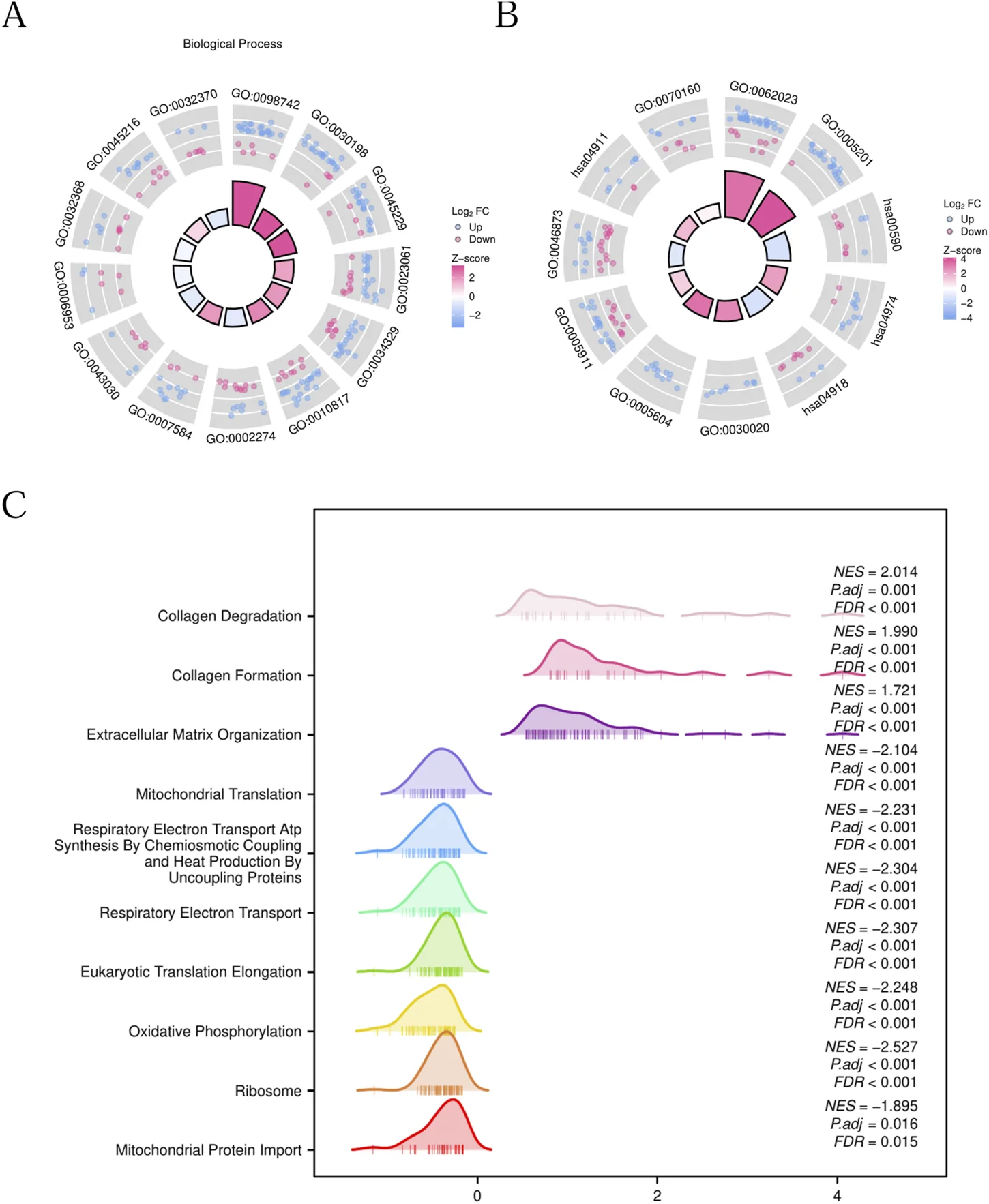
The role of SYTL4 in DLBCL was investigated using gene enrichment analysis. The differentially expressed genes (DEGs) list underwent Gene Ontology (GO) and Kyoto Encyclopedia of Genes and Genomes (KEGG) pathway analyses for clus tering purposes. **(A)** At the biological process (BP) level, 13 pathways were significantly enriched. **(B)** Cellular component (CC), molecular function (MF) and KEGG pathway analysis results. **(C)** The mountain ridge plot displays the gene set enrichment analysis (GSEA) results. FC, fold change; NES, normalized enrichment score; p. adj, adjusted p-value; FDR, false discovery rate.

GO and KEGG analyses of DEGs between the SYTL4 high-/low-expression groups (screening criteria: |log2FC| > 1 with p.adj < 0.05) identified the following findings. At the biological process (BP) level, 13 pathways were significantly enriched (all p.adj < 0.05). Intercellular interaction module: *Cell‒cell adhesion via plasma‒membrane adhesion molecules* (GO:0098742, p.adj < 0.001), *cell junction assembly* (GO:0034329) and *cell‒cell junction organization* (GO:0045216). Microenvironment remodeling module: *Extracellular matrix organization* (GO:0030198) and *external encapsulating structure organization* (GO:0045229). Metabolic regulation module: *Regulation of hormone levels* (GO:0010817), *regulation of lipid transport* (GO:0032368), *positive regulation of lipid transport* (GO:0032370), and *response to nutrients* (GO:0007584). Immune activation module *included myeloid leukocyte activation* (GO:0002274) and *regulation of macrophage activation* (GO:0043030). Acute stress response module: *signal release* (GO:0023061) and *acute-phase response* (GO:0006953) (Figure 4A). Cellular component (CC) analysis revealed significant enrichment in four structures: *collagen-containing extracellular matrix* (GO:0062023, P.adj < 0.001), *cell‒cell junction* (GO:0005911), *basement membrane* (GO:0005604), and *tight junction* (GO:0070160) (the latter three with P.adj<0.05), which may contribute to intercellular interactions potentially relevant to DLBCL. Molecular function (MF) analysis showed significant enrichment in *metal ion transmembrane transporter activity* (GO:0046873, p.adj < 0.05) and *extracellular matrix structural constituent conferring tensile strength* (GO:0030020, p.adj < 0.05), with especially notable enrichment for *extracellular matrix structural constituent* (GO:0005201, p.adj < 0.001). KEGG pathway analysis showed that SYTL4-related genes participate in *arachidonic acid metabolism* (hsa00590, p.adj < 0.05), *protein digestion and absorption* (hsa04974, p.adj < 0.05), and *insulin secretion* (hsa04911, p.adj < 0.05). The significant enrichment of the *thyroid hormone synthesis* pathway (hsa04918, p.adj < 0.05) shows a correlation between SYTL4 expression and endocrine signaling (Figure 4B).

To investigate pathway-level alterations associated with SYTL4 expression stratification, we performed GSEA on the DEGs (p.adj < 0.05, reference group: SYTL4 low expression). The mountain ridge plot (Figure 4C) showed two distinct functional clusters. Patients with high SYTL4 expression presented significant enrichment of extracellular matrix remodeling pathways, including *collagen degradation* (NES = 2.014, p.adj = 0.001), *collagen formation* (NES = 1.990, p.adj < 0.001), and *extracellular matrix organization* (NES = 1.721, p.adj < 0.001). Conversely, SYTL4-low subjects showed enrichment of mitochondrial bioenergetic processes, as reflected by negative enrichment scores in the SYTL4 high-expression group for *mitochondrial translation* (NES = −2.104, p.adj < 0.001), *mitochondrial protein import* (NES = −1.895, p.adj = 0.016), and respiratory chain components: *oxidative phosphorylation* (NES = −2.248, p.adj<0.001), *respiratory electron transport* (NES = −2.304, p.adj < 0.001), and specialized process *respiratory electron transport ATP synthesis by chemical coupling and heat production by uncoupling proteins* (NES = −2.231, p.adj < 0.001). Additionally, the translation-related pathways *Eukaryotic Translation Elongation* (NES = −2.307, p.adj < 0.001) and *Ribosome* (NES = −2.527, p.adj < 0.001) were negatively enriched in patients with high SYTL4 expression.

### Correlation between SYTL4 expression and tumor immune-infiltration in DLBCL

**FIGURE 5:**
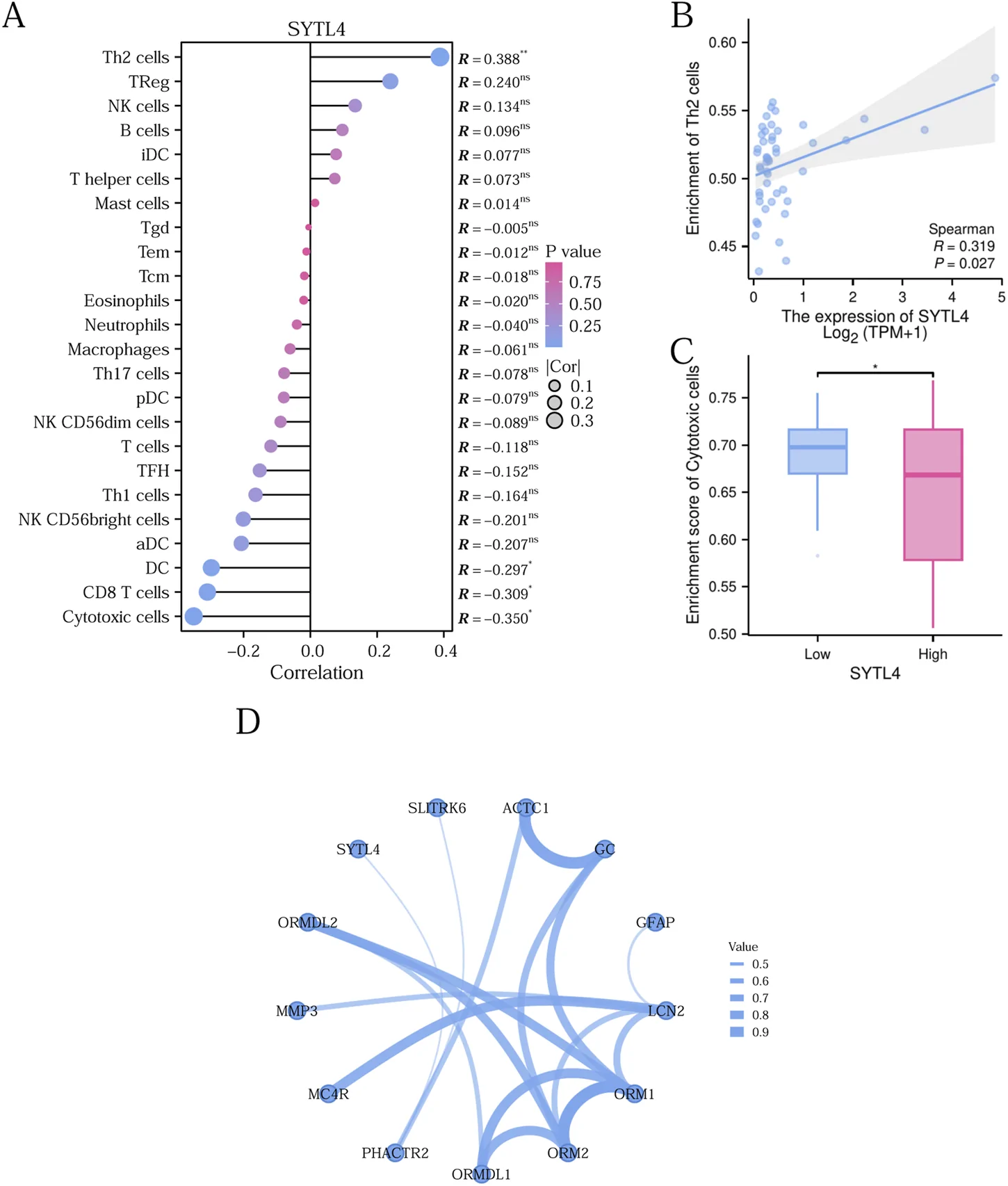
**(A)** Infiltrating immune cells show significant correlations with SYTL4 expression in DLBCL patients, **(B)** particularly Th2 cell associations. **(C)** The high- and low-expression group of SYTL4 exhibited differential enrichment of cytotoxic cells. **(D)** Protein-protein interaction analysis. *P < 0.05; **P < 0.01; ***P < 0.001. SYTL4, synaptotagmin-like 4; Th2, type 2T helper cell; Treg, regulatory T cell; NK, natural killer; DC, dendritic cells; Th1, type 1T helper cell; SYTL4, synaptotagmin-like 4; SLITRK6, SLIT and NTRK like family member 6; ACTC1, actin alpha cardiac muscle 1; GC, GC vitamin D binding protein; GFAP, glial fibrillary acidic protein; LCN2, lipocalin 2; ORM1, orosomucoid 1; ORM2, orosomucoid 2; ORMDL1, ORMDL sphingolipid biosynthesis regulator 1; PHACTR2, phosphatase and actin regulator 2; MC4R, melanocortin 4 receptor; MMP3, matrix metallopeptidase 3; ORMDL2, ORMDL Sphingolipid Biosynthesis Regulator 2.

To explore the potential immunomodulatory role of SYTL4 in DLBCL, we quantified the abundances of 24 immune cell subsets via the ssGSEA algorithm and employed Pearson correlation analysis to investigate their dynamic associations with SYTL4 expression. The lollipop plot showed statistically significant correlations for T helper 2 cells (Th2 cells) (r = 0.388, P = 0.008), CD8^+^ T cells (r = −0.309, P = 0.032), cytotoxic lymphocytes (r = −0.350, P = 0.015), and dendritic cells (DCs) (r = −0.297, P = 0.042) (Figure 5A). Th2 cell abundance was strongly positively correlated with SYTL4, whereas cytotoxic lymphocytes were significantly inversely associated with SYTL4. Furthermore, the scatter plot showed a significant positive correlation between Th2 cells and SYTL4 expression (*P* = 0.027, Figure 5B). The differential immune cell analysis between the SYTL4 high-/low-expression groups showed significantly lower cytotoxic lymphocyte infiltration levels in the high-expression cohort than in the low-expression cohort (*P* < 0.05, Figure 5C), consistent with the negative correlation observed in the Pearson analysis.

### Protein‒protein interaction network

To investigate the functional collaborators of SYTL4 in DLBCL pathogenesis, we constructed a PPI network (Figure 5D). SYTL4 directly interacts with phosphatase and actin regulator 2 (PHACTR2) (combined score = 0.415) (Allen et al., 2004). PHACTR2 emerged as a central hub displaying stronger binding to actin alpha cardiac muscle 1 (ACTC1) (combined score = 0.638) (Kondrashov et al., 2018) and moderate linkage to SLIT and NTRK-like family member 6 (SLITRK6) (combined score = 0.418), a transmembrane receptor (Matsumoto et al., 2011) with oncogenic potential (Morrison et al., 2016). A distinct interaction module was observed involving orosomucoid (ORM) family members (ORM1/2, ORMDL sphingolipid biosynthesis regulator 1 (ORMDL)1/2), with ORM1 exhibiting connections to GC vitamin D binding protein (GC) (Saburi et al., 2017) and lipocalin 2 (LCN2) (Deng et al., 2023).

### Evaluation of SYTL4 expression in tissues by immunohistochemistry

**FIGURE 6:**
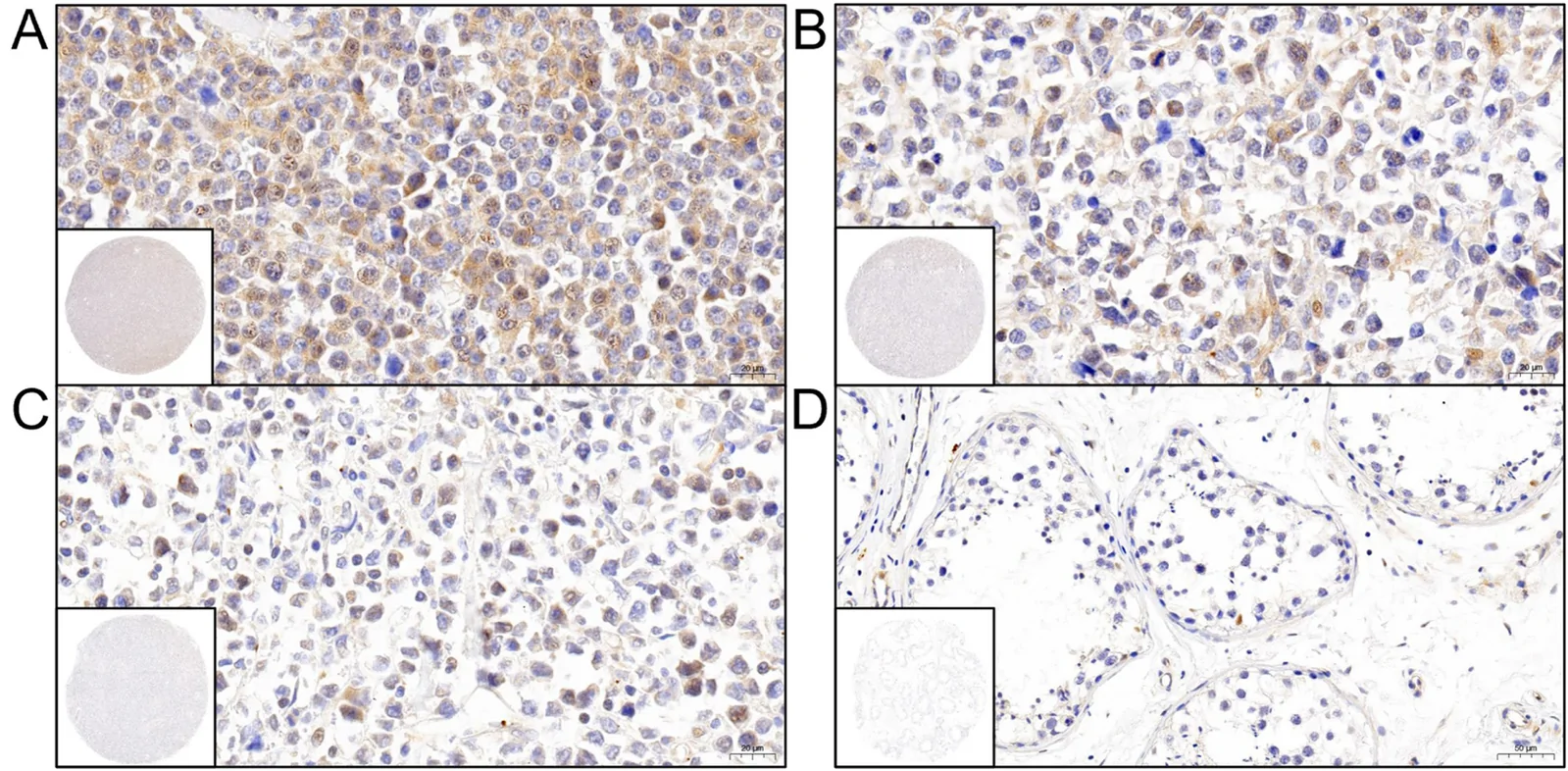
Representative pictures of **(A)** strong, **(B)** moderate, **(C)** weak SYTL4 staining in DLBCL tumor tissues and **(D)** negative in adjacent tissues. Scale bar in a, b, c is 20 μM, and in d is 50 μM.

All 14 tumor samples in the TMA were from male patients, with a mean age of 60 years (range 46–79). Variable levels of cytoplasmic and nuclear SYTL4 staining were observed in the tumor samples. Staining intensity was weak (1+) in 3 (21.4%) cases, moderate (2+) in 5 (35.7%), and strong (3+) in 6 (42.9%) cases. In contrast, both adjacent normal tissues were considered negative (0). Representative photomicrographs are presented in Figure 6.

## Discussion

DLBCL accounts for 30%–40% of all lymphoma cases worldwide (Li et al., 2018). The disease exhibits marked biological heterogeneity, with diverse clinical features and prognostic outcomes, particularly across molecular subtypes (Niroula et al., 2015). Despite advances in therapeutic strategies such as chemotherapy and monoclonal antibodies (e.g., rituximab), approximately 30% of patients experience relapse and poor prognosis (Kwak, 2012). Thus, identifying novel biomarkers and targeted therapies remains critical for improving the survival and quality of life of DLBCL patients.

This study investigated the expression profile and functional role of SYTL4 in DLBCL. Through RNA-seq data analysis and survival assessments, we systematically evaluated the prognostic significance of SYTL4. Our results demonstrate that high SYTL4 expression is significantly associated with adverse clinical outcomes, which is consistent with findings by Kun-Ying et al. in AML (Xie and Wei, 2024). Cox regression analysis confirmed SYTL4 expression as an independent prognostic factor, and a prognostic model incorporating SYTL4 expression effectively predicted patient survival. This model provides a framework for personalized treatment strategies. These findings suggest that SYTL4 may represent a promising candidate biomarker for prognosis evaluation and a putative therapeutic target warranting further investigation in patients with hematological malignancies. In addition, RNA-seq analysis revealed a significant upregulation of SYTL4 expression in DLBCL patients compared to healthy donors, observed in both tissue and peripheral blood samples (Figures 1A,D). ROC curve analyses on two independent datasets demonstrated the diagnostic potential of SYTL4 mRNA levels with certain sensitivity and specificity (AUCs > 0.6), which is consistent with the findings reported by Ren Y et al. (Ren et al., 2025) (Figures 1B,C).This finding was further supported by immunohistochemical staining, which showed higher SYTL4 levels in tumor tissues relative to adjacent normal tissues in testicular DLBCL (Figure 6). Collectively, these findings indicate that SYTL4 not only holds prognostic value but also has the potential to serve as a diagnostic marker.

It is important to note that the diagnostic performance of SYTL4 differed between tissue and blood samples. ROC analysis demonstrated acceptable diagnostic performance in tissue samples (TCGA/GTEx tissue cohort, AUC = 0.949), but lower accuracy in blood (GSE83632, AUC = 0.626), below the conventional 0.7 threshold for a valid diagnostic biomarker (Mandrekar, 2010). This discrepancy likely reflects fundamental differences between sample types: tissue directly captures tumor gene expression, while blood contains nucleic acids from multiple sources and is subject to RNA instability (Lin et al., 2021; Diaz and Bardelli, 2014). Additionally, the limited sample size of the blood cohort (n = 14) may further compromise sensitivity. Thus, while SYTL4 holds promise as a tissue-based diagnostic marker, its application as a blood-based biomarker remains preliminary.

Prognostically, high SYTL4 expression was significantly associated with worse overall survival (P = 0.037) and emerged as an independent prognostic factor (HR = 25.541, 95% CI: 1.776–367.264; P = 0.017). Notably, it did not correlate with conventional clinical parameters such as Ann Arbor stage. This likely reflects the limited sample size (n = 48): categorical stratification rapidly reduces subgroup sample sizes, diminishing statistical power, and the wide confidence intervals of hazard ratios (e.g., SYTL4: 1.776–367.264) further reflect this constraint (Riley et al., 2020). This discordance does not negate the prognostic value of SYTL4; instead, it points to a potential complementary role beyond conventional staging. Nevertheless, these findings remain preliminary and require validation in larger, independent cohorts.

RNA sequencing analysis revealed that high expression of SYTL4 was significantly correlated with 1,074 DEGs, which included 628 upregulated genes and 446 downregulated genes. These findings suggest that SYTL4 may influence the biological characteristics of DLBCL through the modulation of multiple signaling pathways and cellular interactions. This discovery lays the foundation for the development of novel therapeutic targets and highlights the importance of further investigations into the regulatory mechanisms of SYTL4. Functional enrichment analysis revealed that SYTL4-related DEGs were enriched in cell–cell adhesion, ECM organization, arachidonic acid metabolism, and thyroid hormone synthesis, suggesting involvement in tumor cell survival and endocrine signaling. ssGSEA revealed correlations between SYTL4 expression and immune cell infiltration, particularly a positive correlation with Th2 cells and a negative correlation with cytotoxic lymphocytes. This finding implies that SYTL4 may shape the tumor microenvironment by regulating immune cell dynamics, offering insights for immunotherapeutic strategies.

The protein interaction network constructed in this study highlights SYTL4’s correlations with key regulatory proteins in DLBCL. Notably, the direct interaction between SYTL4 and PHACTR2 (Allen et al., 2004), an actin cytoskeletal regulator, suggests a potential link to cytoskeletal dynamics. PHACTR2 interacts with ACTC1 (Kondrashov et al., 2018), an actin-associated protein, further supporting the relevance of cytoskeletal remodeling. This interaction may facilitate malignant cell migration, potentially contributing to disease aggressiveness. Moreover, ORM family members form a distinct interaction module. ORM1 connects to GC (Saburi et al., 2017) and LCN2 (Deng et al., 2023) — proteins known to promote angiogenesis, EMT, and cell invasion—suggesting that SYTL4 could also influence the immune landscape of the tumor (Zheng et al., 2025). Based on this network, we provisionally propose that SYTL4 may contribute to DLBCL progression through two potential axes: cytoskeletal remodeling via the PHACTR2–ACTC1 axis and metastasis-associated processes via ORM family partners (Zhu et al., 2020). We emphasize that these proposals are derived from enrichment analysis and remain hypotheses; experimental validation (e.g., gene silencing or knockout) is necessary to confirm their functional relevance.

Several limitations should be noted. First, the present study is primarily based on public RNA-sequencing datasets and lacks comprehensive protein expression and functional validation, which represents a major limitation for confirming the predicted mechanisms (e.g., the PHACTR2-ACTC1 axis and ORM-associated pathways). Furthermore, the correlation of SYTL4 with immune cell infiltration (e.g., Th2 and cytotoxic cells) was assessed only at the transcriptomic level, and direct validation in DLBCL tissues at the protein level has not been performed. Second, the small TCGA cohort (n = 48) prevented robust evaluation of SYTL4 associations with molecular subtypes (e.g., the LymphGen classification: MCD, BN2, N1, EZB, ST2, A53 (Wright et al., 2020)), due to missing subtype annotations and limited statistical power (∼8 cases per subtype). Third, IHC validation was restricted to a small testicular DLBCL cohort (n = 14) with narrow age range (mean 60 years) and no female representation, precluding assessment of sex/age effects and limiting generalizability. The limited sample size also precluded analysis of the correlation between SYTL4 protein expression and clinicopathological parameters or patient survival, which would be necessary to validate its clinical relevance. Anatomical homogeneity and potential batch effects across datasets further constrain consistency. Future larger, multi-center cohorts with diverse subtypes, sex/age balance, standardized molecular subtyping, and functional assays are needed to validate SYTL4’s prognostic and therapeutic value.

## Conclusion

In summary, this study identifies SYTL4 as a potential key regulator and prognostic biomarker in DLBCL. Our findings offer novel molecular insights and a foundation for future mechanistic studies. However, as the current evidence is primarily bioinformatics-based, experimental validation is required to confirm its regulatory role.

## Data Availability

Publicly available datasets were analyzed in this study. This data can be found here: RNA-seq data for 33 TCGA cancer types and matched GTEx normal tissues were obtained from University of California Santa Cruz XENA (UCSC XENA, https://xenabrowser.net). The DLBCL-specific RNA-seq data (STAR-generated counts) and clinicopathological records of 48 patients were downloaded from the TCGA portal. In addition, we incorporated the GSE83632 dataset from Gene Expression Omnibus (GEO, https://www.ncbi.nlm.nih.gov/geo/) for analysis.

## Glossary

ACC: adrenocortical carcinoma
ACTC1: actin alpha cardiac muscle 1
AML: acute myeloid leukemia
AUC: area under the curve
BLCA: bladder urothelial carcinoma
BRCA: breast invasive carcinoma
BSA: bovine serum albumin
CESC: cervical squamous cell carcinoma
COAD: colon adenocarcinoma
DAB: diaminobenzidine
DCs: dendritic cells
DEGs: differentially expressed genes
DLBCL: Diffuse large B-cell lymphoma
EMT: epithelial-mesenchymal transition
ESCA: esophageal carcinoma
FDR: false discovery rate
FFPE: formalin-fixed paraffin-embedded
GBM: glioblastoma multiforme
GC: GC vitamin D binding protein
GEO: Gene Expression Omnibus
GO: Gene Ontology
GSEA: Gene Set Enrichment Analysis
GTEx: Genotype-Tissue Expression
HNSC: head and neck squamous cell carcinoma
KEGG: Kyoto Encyclopedia of Genes and Genomes
KICH: kidney chromophobe
KIRC: kidney renal clear cell carcinoma
KIRP: kidney renal papillary cell carcinoma
LCN2: lipocalin 2
LGG: brain lower grade glioma
LIHC: liver hepatocellular carcinoma
LUAD: lung adenocarcinoma
LUSC: lung squamous cell carcinoma
MSigDB: Molecular Signatures Database
NHL: non-Hodgkin lymphoma
ORM: orosomucoid
ORMDL1: ORMDL sphingolipid biosynthesis regulator 1
OS: overall survival
OV: ovarian serous cystadenocarcinoma
PAAD: pancreatic adenocarcinoma
p.adj: adjusted p-value
PBS: phosphate buffer saline
PD: progressive disease
PHACTR2: phosphatase and actin regulator 2
PPI: protein-protein interaction
PRAD: prostate adenocarcinoma
READ: rectum adenocarcinoma
ROC: receiver operating characteristic
SD: stable disease
SKCM: skin cutaneous melanoma
SLITRK6: SLIT and NTRK like family member 6
STAD: stomach adenocarcinoma
SYTL4: synaptotagmin-like 4
TCGA: The Cancer Genome Atlas
TGCT: testicular germ cell tumors
Th2 cells: T helper 2 cells
THCA: thyroid carcinoma
THYM: thymoma
timeROC: time-dependent receiver operating characteristic
TMA: tissue microarray
TME: tumor microenvironment
TNBC: triple-negative breast cancer
UCEC: uterine corpus endometrial carcinoma
UCS: uterine carcinosarcoma
UCSC XENA: University of California Santa Cruz XENA
US: United States
